# **Situation Analysis of R & D Activities: An Empirical Study in Iranian Pharmaceutical Companies**

**Published:** 2012

**Authors:** Hamid Reza Rasekh, Gholamhossein Mehralian, Abbas Ali Vatankhah-Mohammadabadi

**Affiliations:** a *School of Pharmacy Department of Pharmacoeconomics and Pharmaceutical Management, Shahid Beheshti University of Medical Sciences, Tehran, Iran. *; b *Pharmaceutical Sciences Research Center, Shahid Beheshti University of Medical Sciences, Tehran, Iran.*; c *Student Research Committee, Shahid Beheshti University of Medical Sciences, Tehran, Iran. *

## Abstract

As global competition intensifies, research and development (R & D) organizations need to enhance their strategic management in order to become goal-directed communities for innovation and allocate their resources consistent with their overall R & D strategy. The world pharmaceutical market has undergone fast, unprecedented, tremendous and complex changes in the last several years. The pharmaceutical industry is today still one of the most inventive, innovative and lucrative of the so-called “high-tech” industries. This industry serves a dual role in modern society. On one hand, it is a growing industry, and its output makes a direct contribution to gross domestic product (GDP). On the other side, drugs, this industry’s major output, are an input in the production of good health. The purpose of this study is to evaluate R & D activities of pharmaceutical companies, and also to highlight critical factors which have influential effect on results of these activities. To run this study a valid questionnaire based on literature review and experts’ opinion was designed and delivered to 11 pharmaceutical companies. Empirical data show there is not acceptable situations considering of the factors that should be taken in to account by managers including; management commitment, human resource management, information technology and financial management. Furthermore, we concluded some interesting results related to different aspects of R & D management. In conclusion, managers must be aware about their performance in R & D activities, accordingly they will able to take a comprehensive policy in both national and within the company.

## Introduction

Pharmaceutical research and development (R & D) is the process of discovering, developing and bringing to market new ethical pharmaceutical products. Industrial research and development is a scientific and an economic process. Science specifies the opportunities and limitations, but economics determines which opportunities and scientific challenges will be addressed through industrial investigation (1).

If a pharmaceutical company wants to achieve market success with a new product brand, it needs to invest seriously on marketing and sales practices. Therefore, not surprisingly, we may come into this result that basic research and development together with marketing and sales practices are two of the most important practical and even more strategic priorities of the world pharmaceutical industry. Inevitably, the greatest investments of the pharmaceutical industry are done in all terms. So, innovator pharmaceutical companies through the world, approximately 16% of their sales on R & D and even more, about 26% or more on marketing and sales practices, on average (2).

The pharmaceutical industry performs a dual role in modern society. On one hand, it is a developing industry and its output makes a direct cooperation to gross domestic product (GDP). On the other hand, prescription medicines, the major yield of this industry, are an input for producing good health. These products make an important cooperation to improve the population health as well as life expectation. Societies are interested in prescription medicines and pharmaceutical innovation in large part because of the potential health advantages which are obtained from medicines. But particularly in light of increased competition over the world, many countries are searching sectors in which they have a comparative advantage. Pharmaceuticals and biopharmaceuticals assumed to be more desirable in many countries which have higher incomes because of their reliability on high educated working forces and production may be less probably to be outsourced to countries in which common salaries are lower. This dual role has greatly increased the complexity of public-sector policy making (3).

The world pharmaceutical industry greatly has been changing in the last decade. Serious globalization, increased competitiveness and the fight for worldwide market shares which develop new challenges for pharmaceutical companies. Fast globalization certainly empowers the integration of pharmaceutical industry of the world. Coherence in forms of mergers and alliances prevail more and more as a strategic orientation for the world pharmaceutical companies. By coherency, they are going to develop strategic cooperation in order to be successful, competitive and continuous development cycles (2).

Global competitiveness is becoming increasingly important for the pharmaceutical industry. Companies are seeking for opportunities to increase the performance of the resources for benefit at all phases of the overall value chain from discovery research to production and logistics as well as sales and marketing issues. Particularly innovation is recognized as the infrastructure for competitive advantage and developed by strong investments in R & D (4). But drug development and commercialization is a costly, time consuming and hazardous process. Global studies which are published in 2003 report an average pre-tax cost of approximately US$800 million to bring a new medicine to the market (5, 6). It is estimated that when a novel drug is released on the market, an average of 12-13 years would have been passed since the synthesis of the new active pharmaceutical ingredient (API). Thereby, on average, out of every 10,000 materials synthesized in laboratories, only one or two will successfully pass all the phases to be marketable medicines (7). In developing countries, according to the lack of economic motivations, the pharmaceutical industry usually doesn’t invest on novel API. Moreover, according to low capacity of government for covering the cost of these prices of the pharmaceutical innovations, these innovations are limited. 

According to literatures, the evaluation of R & D activities in industrial firms has investigated the issue from different perspectives. So, they have concentrated on some remarkable perspectives (8-10). Considering that the number of relevant contextual factors in this topic, we decided to confer a more specific and definite scope to our research. So far, in Iran, no scientific study has been conducted about assessment of current situation of R & D activities in pharmaceutical companies. Present study based on aforementioned critical factors, proposed the research question as follows:

(I): “How is current situation of R & D activities in pharmaceutical companies?”

To answer this question, there are some essential sub-questions which lead to find the final answer.

“How is current situation of management commitment about R & D activities in pharmaceutical companies?”

“How is current situation of human resource management about R & D activities in pharmaceutical companies?”

“How is current situation of financial management about R & D activities in pharmaceutical companies?”

“How is current situation of information technology (IT) management about R & Dactivities in pharmaceutical companies?”

(II): “What is managers’ attitude about influencing factors in R & D activities? 

To present the paper, this article is divided into two substantial sections, the first section depicts the summarized outline of the pharmaceutical industry particularly in Iran, it also incorporates literature review and research methodology of this study and the second section deals with analysis of the collected data besides conclusions and implications of the this study.

*Literature review*

*Iranian pharmaceutical over review*

Medicine and pharmacy are among the oldest sciences and disciplines in Iranian civilization. After Islam was introduced to Iran, it had a great impact on both sciences. The influence was so great that it drew a line in the history of pharmaceutics in Iran. There are two different but continuous eras of medicine and pharmacy of Iran; before Islam and after Islam. The sciences of medicine and pharmacy were greatly improved during the reign of Islamic civilization. The Islamic pharmacists and physicians followed methods of Hippocrates and Galen. Among the most famous Persian physicians and chemists are Mohammad-ebn-e ZakariaRazi and Avicenna who both were living during Medieval era. The most popular book of Avicenna in medicine is “Ghanoon” written in five volumes. Two volumes of the book are devoted to pharmacology (11). 

*Pharmaceutical companies in Iran*

On the eve of the 1979 revolution, numerous domestic, foreign, and domestic-foreign private companies were active in Iran’s pharmaceutical sector. By that time, the country’s pharmaceutical sector had been transformed into a market that boasted a $300 million annual cash flow. There were nearly 4,000 kinds of pharmaceutical products available in Iran, 70% of which was provided by imports and the remaining 30% was produced domestically. More than half of the latter market served the sales of products under the concession of foreign companies (12). Now more than 95% of the drug consumption is produced by domestic pharmaceutical companies (13). 

*Generic System in Iran*

**Figure 1:**
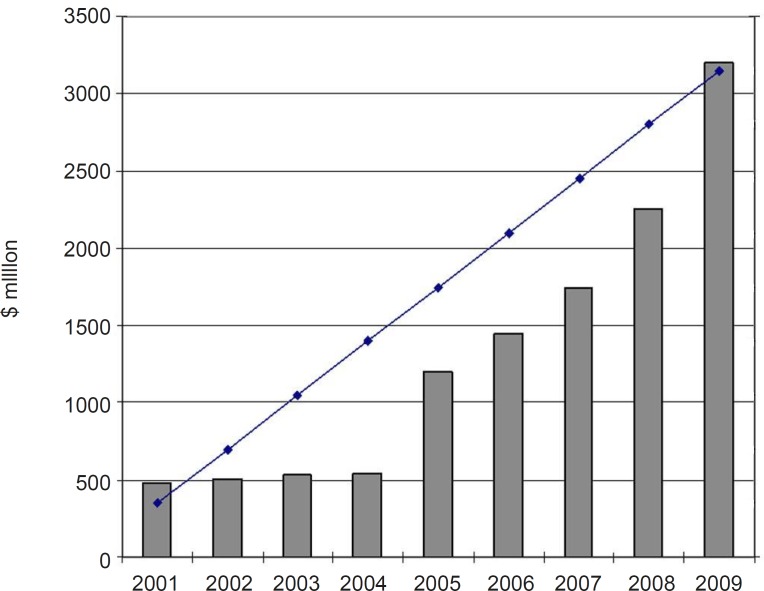
Iranian pharmaceutical market (16).

The year 1981 witnessed the beginning of a roundup of actions aimed at adopting and implementing policies to modernize the Iranian pharmaceutical sector, which influenced this industry all the way up to 1994. These programs, entitled Generic Scheme, sometimes also called the Generic Concept, formed the foundation of the new pharmaceutical system in the country. In recent years, national pharmaceutical system was directed to the brand-generic and brand systems and, as a result, there is some competition in the industry. This provides good opportunities for future development of domestic pharmaceutical industry. The fact is that the domestic industry has not yet adequately developed to its full capacity and there are much potential capabilities for further growth and development. Domestic pharmaceutical industry is experiencing a substantial double digit growth in the recent years. Furthermore, in house production of hi-tech biological products is an emerging know- how in Iran’s pharmaceutical sector. In recent years some private firms have focused to produce biological pharmaceuticals, using novel biotechnology methods (14). The annual growth of Iranian pharma market value (2001-2009) is shown in Figure 1. The share of domestic pharmaceutical sale to total pharmaceutical sale in the year 2009 was around 60 percent (15). 

The entire R & D process is a highly regulated sequential procedure, starting with the so called research stage that covers the biological validation of the drug targeted and the subsequent chemical optimization of the potential drug candidate. Moving forward to early development, preclinical phase mainly comprises animal testing. Before entering the clinical phases an investigational new drug application must be submitted to the regulatory authorities. Following the previous stage, the compound is administered to healthy volunteers in clinical phase I to gather information about safety and dosage. In clinical phase II, application to a small number of patients is done to obtain proof of the concept. The next step of late development is characterized by clinical phase III studies that include a larger number of patients to ensure statistical significance. After successful completion, a new drug application is submitted to the regulatory authorities to be eventually admitted for market launch of the product candidate (17). 

If a pharmaceutical company wants to reach market success with a new product, it needs to invest seriously on marketing and sales practices. Therefore, not surprisingly, we may reach to this result that basic research and development (R & D), together with marketing and sales practices are two of the most important practical and even more strategic priorities of the world pharmaceutical industry. Approximately, world pharmaceutical companies invest 16% of their sales on R & D and even more, about 26% or more on marketing and sales practices. However, these proportions, especially the one of R&D investment, are even higher with the proficient, like biotechnology and pharmacogenomic companies, and much lower with the typical pharmaceutical companies. As stated, the world pharmaceutical industry structurally is not unique and pharmaceutical companies are varies according to their infrastructural mission, efficiency and strategic development. The world pharmaceutical companies could be categorized into three different classes (2); pharmaceutical companies which first and foremost are work on infrastructural research, development and marketing and sales of new brand, innovative, original pharmaceutical products (called originators); pharmaceutical companies which first and foremost are work on development and sales of generic products (called generic producers); and pharmaceutical companies which first and foremost are work on infrastructural research and development of biopharmaceutical and pharmacogenomic products and technologies of new delivery systems (called proficiencies). 

As we are going to develop new product, changing the features of the world pharmaceutical industry environment is important issue which should be considered primarily including: increased globalization, changing structure of competition and increased competitiveness, lack of new products, increased investments on R & D practices, fast integration and focus of the world pharmaceutical industry, increased importance of strategic management, development of new remedy fields and technologies (biotechnology, pharmacogenomics), ageing of world population and introducing of new, not yet desirably covered remedy fields, and finally, fast development of the globe generic markets (18).

*Key factors in R & D practices*

According to extensive literature review, some of critical factors which have substantial effect on outcome of R & D activities and potentiality affect performance of R & D activities were obtained, they are discussed as follows:

*Management commitment*

World teacher of management, Peter Drucker, who has particularly emphasized the core importance of strategic marketing management for a successful and long-term highly competitive business efficiency of companies, has once said something about globalization and globalization management: “In the upcoming years, there will be two kinds of top managers: those who would be able to think globally with strong strategic marketing management engagement and those jobless”. Strategic marketing management with an emphasized global method of thinking, performance and management thus enables companies to put customers into the center of all their business practices and integrally concentrated all business practices to a common and final target – to be successful in satisfying customers’ needs and to be better than the competitors (2). However, changes provide new challenges as well as develop new business opportunities. Thus, it is important to react and act quickly and to be proactive. 

Success from innovation in the pharmaceutical industry is affected by a number of externalities containing universities, the government, financial institutes, local worker markets and industrial connection co-operators (19). These factors provide particular knowledge at multiple points in the value chain. Universities provide access to outstanding scientists, an easily available source of scientific labor and intellectual property (20). Industrial co-operators support investigating and utilization practices such as R & D, product development, FDA confirmation and sales through strategic connections (21, 22). The reasons offered for inter-company strategic connections in the pharmaceutical industry are as follows: access capitals for R & D, the decrease of risk in R & D, quality control in R & D, product development and producing for large scale researches, improvement of trustworthiness and reputation, marketing and distribution and acquiring the attention of third party investors (*e.g*. risk capital) (19). The key components in this business are knowledge, money and organizational support. The government, industry, and academic environments are joining together to improve the regulatory structure, industrial capacity and man powers that will be required. That supporting environment has been developed and backed by a science-based regulatory framework and fast growing industrial capacity and man powers (23).

It is thus clear that some pharmaceutical companies are not able to satisfy long-term and ever-changing market needs and customers’ expectations, to invest heavily into R & D and marketing and sales activities in the endeavor to bring new products to the markets properly. It can clearly be seen that this process enables the pharmaceutical companies for new development circles and their long-term development and growth. Strategic activities like alliances for maintaining long-term growth and competitiveness is today one of the most usable strategies in the world pharmaceutical industry. It can be argued that pharmaceutical companies make alliances in endeavors to create common synergies and to better exploit their common assets, knowledge, product life cycle and, moreover, to improve their strategic market positions. Thus, the most important and strategic activities of creating common strategies for the pharmaceutical companies are: (i) products, to gain market shares and to drive the sales growth; (ii) research and development (R & D), to create new products; (iii) markets, to create geographic and market expansion; (iv) marketing and sales activities, to compete on the global markets and finally (v) financials, to create common cost reduction synergies and investment capabilities (2).

*Human resource management*

The competitive position of leading pharmaceutical companies is strongly dependent on the ability of R & D scientists to develop new medicines. Certainly, within the industry there are very close connections between business success and scientific success. Therefore, scientific employees have been central to the achievements of the main pharmaceutical companies over the recent 10-15 years (24). There has also been a general move to give scientists a keener awareness of the commercial realities of R & D, although the efficiency of these initiatives is questionable. Accordingly, the human resource specialists understand the importance of employing R & D personnel with a strong engagement to science (24).

In the pharmaceutical industry, star scientists provide crucial connectivity to universities and other sources of upstream knowledge (25). Star scientists are important border spanners, because a difference in coding systems exists, specifically between large public institutes and academic institutions and other high-technology beginners. This mismatch develops the possibility of communication problems (26). It can be moderated, however, by the use of persons ‘who are capable of translating between two coding systems either by personal contact or knowledge of the literature, and who can act as bridges linking the organization to other organizations and workers in the field’ (26). The variations in coding systems between different knowledge communities is of concern because without border spanners that acted as translators, the institute would potentially be unable to incorporate tacit information into codified knowledge that can lead to future innovation (27).

In addition, in high-speed knowledge environments, border spanners are not only able to keep the pulse of shifts in technology, but in many cases may actually hold main knowledge themselves (27). Considering the important role of manpower in this environment, accordingly, the relationship between industry and academic center considerably should be considered especially by governmental policy.

*Information technology (IT) management*

Koenig’s research discovered that the more successful pharmaceutical companies showed greater openness to information, were less involved in protecting proprietary information, had a greater development of information systems, promoted use of information (including searching and serendipity) and taken benefit from greater technical and subject experience in their information (28). The pharmaceutical industry is a knowledge-based industry which shares many of its features with classical technology-focused industries, *e.g*. R & D severity and focus and the use of new scientific concepts (29, 30), but there are also some unique specifications such as the highly-regulated environment, the long development cycle and a high level of risk and cost during the research process (31). The R&D site is dependent upon applied research within many systems: medicine, organic chemistry, biochemistry, and pharmacology and medicine delivery. Work on a new medicine is often performed at a number of laboratories located in different countries (32). They are many systems need to work together in an efficient way in order to develop and share the knowledge leading to a new medicine (33). 

*Financial resource management*

Research and development for medicines is a time and cost-consuming and costly process. Although there is no general agreement about how to compute the cost for research and development in the pharmaceutical market and on the actual size of the cost, there is a general agreement that the mean R & D cost is accompanied with introducing a new prescription medicine is in the scope of hundreds of millions of dollars (6, 34). The high cost is assignable in part to the explicit criteria for medicine confirmation set by government agencies (35, 36). In most countries governments bound producers to display both safety and efficacy before a medicine can be approved for sale. Consequently, medical researches for effeicacy and quality control have become the most costly process in the development of a new medicine (3). 

**Figure 2:**
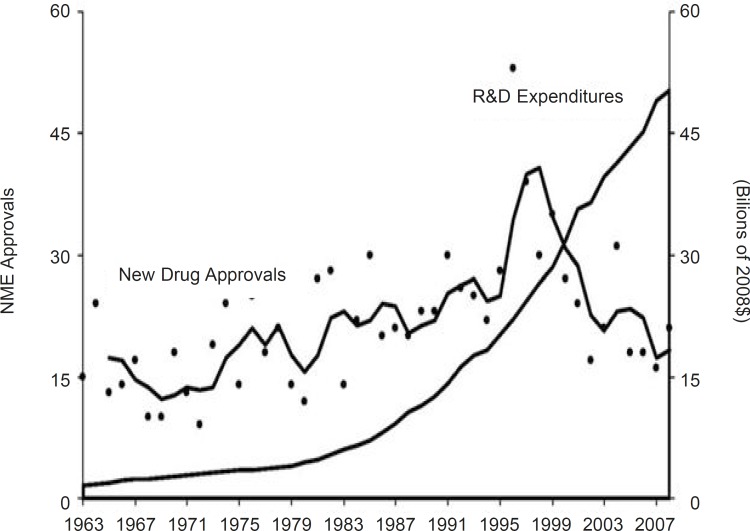
New drug approvals and R & D spending (37).

Such researches and development for new medicine is a time-consuming and costly process and is globally accepted. Pharmaceutical institutes typically allocate a comparatively considerable share of their resources on R & D in comparison to do their counterparts in most other industries or relative to spending by the public sector. As instance, R & D spending as a percentage of GDP ranged from 2% to 3% in major developed countries such as United States, Japan, Germany and France during 1988–2002. By contrast, pharmaceutical producers of monopolized medicines consistently spent over 10% of their sales income on R & D during the recent two decades (3). Accordingly, as shown in figure 2 the scope of R & D expenditures and new medicine approval rate in this industry weren’t in same way (see Figure 2). 

If producers sell medicines at the incremental cost of production and distribution, they would have no way to pay back the cost of R & D. To pay back such cost, in most countries monopolies give market power to new products and exclusiveness profits provide the cash flow to cover and develop a return on investments in R & D. However, the monopoly order is a double-edged: it protects the motivation for R & D on one hand, but on the other hand it develops entrance border in the pharmaceutical market. This entrance border results in higher prices and costs as well as lower levels of use of the new medicines that would be common under competition. This has led to some specialists to suggest substitutes to the monopoly order, such as monopoly buyouts and pre-payment mechanisms that would keep the motivation for product development (3). 

*Study design*

In this section we provided a methodology for operationalizing the variables and factors, acquiring the data and determining the reliability of factor grouping. The data used in this study gathered from the questionnaire distributed to managers of 11 Iranian pharmaceutical companies. In order to evaluate R&D activities, the questionnaire is designed in two sectors; ones evaluate the current situation of R&D management and the second sector tends to determine attitude of participants according four aforementioned factors. The four critical factors which listed in Table 1, including: management commitment, human resource management, IT management and financial management, in the second sector the chosen response could be strongly disagree, disagree, no opinion, agree and strongly agree. In addition to the above questions, information related to the basic profile of the interviewees was requested at the end of the questionnaire. The main sampling targets were senior managers or R & D department managers.

**Table 1:** Critical factors and questions

**Factor dimension**	**Questions **	**researchers**
Management commitment	• Existence of R&D department • Supporting system for ideas and creativity • Codified documentations • Documented strategy for R&D activities	Herdman GC.1993 (1), Kesič D. 2009 (2), Bagchi-Sen S. 2007 (19), Kenney M. 1998 (20), Powell WW, Koput KW, Bowie JI and Smith-Doerr L. 2002 (21), Prevezer M. 2001 (22), Mao D and Zheng Q. 2009 (23).
Human Resource Management	• Domestic human resource • Creativity test for recruitment • Promotion of R&D employee • Difference in salary between R & D sector and others • Documented method to compensation of R & D employee • Incentives for training • Facilities for attending to domestic seminars • Facilities for attending to external seminars • Documented training program • Journal club for R & D employee • Personnel involvement in strategy formulation	Jones O. 1996 (24), Arora A and Gambardella A. 1990 (25), Allen T and Cohen SI. 1969 (26), Hess AM and Rothaermel FT. 2011 (27).
IT Management	• Accessibility to library • Accessibility to patent resource • Project managers	Wang MY. 2006 (28), Pisano G. 1997 (29), Santos F. 2003 (30), Cardinal LB. 2001 (31), Omta S, Bouter L and Van Engelen J. 1997 (32), Omta S and Van Engelen J. 1998 (33).
Financial Resource Management	• Project cost and budget allocation • Benefits from Pharmacoeconomics studies • Allocated budget to R & D activities • Enough investment in R & D activities • Screening or Exploratory test besides QA* and QC*	Kesič D. 2009 (2), Sloan FA and Hsieh CR. 2007 (3), DiMasi JA, Hansen RW and Grabowski HG. 2003 (6), Light DW and Warburton RN. 2005 (34), Grabowski H, Vernon JM and Thomas LG. 1978 (35), Cockburn I and Henderson R. 2001 (36), Grabowski H. 2011 (37).

*Note: QA: Quality assurance; QC: Quality control

*Reliability and Validity of the questionnaire*

The internal consistency of a set of measurement items refers to the degree to which items in the set are homogeneous. Internal consistency can be estimated using a reliability coefficient such as cronbach’s alpha (38). In this research cronbach’s alpha was calculated 0.88. The validity of a measure refers to the extent to which it measures what is intended to be measured. Content validity is not evaluated numerically, it is subjectively judged by the researchers (39). To gauge the acceptance of the questionnaire, 10 people who qualified in field of pharmaceutical practice were invited to participate in a pilot test. The participants suggested adding and omitting some parts of questionnaire. Finally, all the pretest participants expressed strong agreement with the suitability of the questionnaire. The questionnaire was considered finalized after modifying some of the questions.

*Data collection*

Data for this study have been gathered using questionnaire that was distributed to 11 pharmaceutical firms which affiliated to two large holding companies in Iran. In order to understand the viewpoint on R&D activities from key sector of the pharmaceutical industry, questionnaires were sent to the different main department besides research and development. Accordingly, respondents from managers who had comprehensive knowledge about company’s process, R & D activities and general pharmaceutical related issues were selected. The number of questionnaires sent out was 95; the number returned was 72 with a return rate of 75.6 percent. *Survey results and analysis*

In this study data were collected using a self-administered questionnaire that was distributed to 2 big holding companies which include 11 pharmaceutical companies. Questions also included demographics profile such as educational level, position and gender which are shown in Table 2. The SPSS software as common tool was used in the current paper.

**Table 2:** Demographics of the respondents

Gender				Position		Educational Level		
	Frequency	%		Frequency	%		Frequency	%
Male	54	75	Managing Director	10	13.9	BS	4	5.6
			R&D Manager	14	19.4			
						MS	4	5.6
			Production Manager	10	13.9			
Female	18	25				PharmD	56	77.8
			Technical Responsible	13	18.1			
						PhD	7	9.7
			Managerial Board	21	29.2			
Missing Total	72	100.0		4 72	5.6 100.0		1 72	1.4 100.0

As shown in Table 3, there are 20 questions about current situations of four major factors in R & D activities. It can be seen intriguing findings related to some questions, for example around 83 percent of participants claimed that there is not difference in basic salary among R & D department with other departments, and according to documented policy for compensation of new contribution presented by employee as formula or patent registration, around 63 percent asserted there is no strategy for that. Related to documented policy in budget allocation for R & D activities, 60 percent participants selected NO option.

**Table 3:** Questions related to current situations

**Questions**	**Yes**	**No**	**Questions **	**Yes**	**No**
Do you have R & D department in your company?	95.6	2.2	Is there any policy for detecting of novel ideas?	55.6	42.2
Do you have problem as lake of domestic researcher for recruitment?	60.0	35.6	Do you have routines for documentation of research projects?	75.6	20.0
Do you use determinant test as extent of creativity for recruitment?	37.8	57.8	Do you have a separated space as library in your company?	44.4	48.9
Do you have nonfinancial incentive policy for your R & D department?	35.6	62.2	Do you have accessibility to in house or foreign patents?	13.3	75.6
Is there difference in basic salary among R & D department with other departments?	15.6	82.2	Do you have a responsible person or department for controlling of projects progress?	66.7	28.9
Do you have documented policy for compensation of new contribution as formula or patent registration?	33.3	62.2	Do you have routines for calculating of projects costs or budget allocation?	66.7	33.3
Are there any incentive programs for attending of R & D personnel in educational and training courses?	44.4	51.1	Do you use pharmacoeconomic analysis for delivering your novel projects?	44.4	46.7
Is there any financial support for attending in domestic scientific conventions?	68.9	28.9	Do you have document policy in budget allocation for R & D activities?	26.7	60.0
Is there any financial support for attending in abroad scientific conventions?	48.9	48.9	Do you have documented strategy for R&D activities?	40.0	53.3
Is there documented educational and training program for R & D personnel?	62.2	33.3	Is there scientific workshop like journal club particularly for R & D personnel?	2.2	95.6

*Analysis of attitude survey*

*T- test analysis*

In order to analyze respondents’ attitude according to critical factors, in the first step T- test analysis has been done. Table 4 shows the result of t-test, exception IT, all of the factors have significant and positive difference with cut point 3. It means that factors including management commitment, human resource and financial resource should be taken into account by managers and directing board to achieve R andD goals.

**Table 4:** Result of mean difference (one sample t- test).

**Critical Factors**	**Mean**	**Std. Deviation**	**T Statistic**	**Sig. Level**
Management commitment	3.61	0.31	13.45	.000*
Human resource	4.06	0.45	16.27	.000*
IT	2.91	0.63	-0.933	.355
Financial resource	4.29	0.39	22.75	.000*

Notes: Significant at *0.05

*Correlation analysis*

Pearson correlation to test the relations among critical factors which means what›s the inter correlation among basic factors were used. In this study, the results indicated that the critical factors have partially correlated together. 

As seen in the Table 5, management commitment has positive and significant correlation with human and financial resource and IT show positive and significant correlation with financial resource. But, there is no significant correlation between management commitment and IT, and between financial resource and human resource.

**Table 5:** Correlation results

	**Management Commitment**	**Human Resource**	**IT**	**Financial Resource**
	Pearson Correlation	Pearson Correlation	Pearson Correlation	Pearson Correlation
Management commitment	1	**0.550	153.	**514.
Human resource	**550.	1	234.	343.
Information technology	153.	234.	1	**650.
Financial resource	**514.	343.	**650.	1

**Notes**: Correlation is significant at the **0.05 level

*Friedman test results*

According to Table 5, Friedman test result shows that the human resource has the highest priority in compared with others factors according to respondents’ attitude.

## Discussion

If a pharmaceutical company wants to achieve market success with a new product, it needs to invest strongly into marketing and sales activities. Thus, not surprisingly, we may conclude that basic research and development (R & D), together with marketing and sales activities are two of the most important operative and even more strategic priorities of the world pharmaceutical industry. Approximately, world pharmaceutical companies invest 16% of their sales into R & D and even more, about 26% or more into marketing and sales promotions (2, 40). The logical correlation between R & D success and some critical factors has encouraged us to run present study. In this study, we have tried to evaluate R & D activities among Iranian pharmaceutical companies. Accordingly, this study has focused on critical factors which have potentiality to make a reliable structure for R & D department.

As shown in Table 4, t-test results based on respondents’ attitude indicated that difference between four factors and cut point (3) were significant, but there is just one exception related to IT. There are several studies which argued by researches that these factors are fundamental to achieve valuable goals in R & D activities. Considering the human resource, for example, such studies (41-43) have emphasized the need to contract people with greater technical and scientific knowledge in these tasks. According to managerial support, our result is consistent with previous evidences (44-46). Although IT can strongly promote efficiency of R & D activities through increasing of knowledge sharing among employees, there is a weak attitude toward it according to findings of this study.

We also have some statistical findings which can help us to understand better situation of R & D activities in Iranian pharmaceutical industry. Results show that maximum ratio of R & D employees to total employees is 0.03, and also 75.6% of them were Pharm D, while 13% has PhD level. The experts who graduated from chemistry comprised around 40% R & D employees.

Considering the existence of library and scientific documentation as an important component of structural (organizational) capital (16) which has influential impact on knowledge sharing, 47% of respondents depicted that there was no library in their companies. Technology transferring from academic or research centers as a critical factor in pharmaceutical environment in order to gain competitive advantages has not effectively been considered among pharmaceutical managers in Iran. Because, answers show that approximately 40% of evaluated companies didn’t pay attention to aforementioned issue during 2011, as Subbanarasimha *et al. *(2003) pointed out that among the resources which a firm uses, technology transferring is an imperative one as it can help firms both attain and sustain their competitive advantage (47).

Findings show that managers have weak attitude toward on-job training of R & D employees due to non considerable financial allocation to this issue, since basically Katsanis (2006) pointed out that continuous training program is a key tool for employees and managers performance. The other explanation may be associated with a weak relationship between industry and academic center, while Fontana *et al. *(2006) believed that this relationship is extensively important for pharmaceutical companies (48, 49).

Considering the key role of financial resource for R & D activities, respondents answered that only 37% of them have a documented budget allocation to their R & D activities, but it is not in turn with literatures, so argued both R & D and sale activities comprise two the highest investment in pharmaceutical practice. Recently, outsourcing policy among pharmaceutical companies has became well known strategy to overcome the lack of financial resources and to fundamentally reduce costs (50), but findings show that around 40% of evaluated companies didn’t attention to this issue and surprisingly 42% claim that they just have 1-2 outsourcing projects during 2011. Furthermore, we can see also a weak motivation toward co-development of products and delivering novel medicine to society; while some similar countries to Iran (*i.e*. India, China, Turkey and Jordan) have paid a lot of attention to this powerful strategy in last decade, and as a result they could deliver new medicine at a reasonable price to the market (40). 

**Table 6:** Results of Friedman test

**Critical Factors**	**Mean Rank**
Management commitment	1.99*
Human Resource	3.12*
IT	1.23*
Financial Resource	3.67

Notes: Friedman test is significant at the* 0.05 level

Considering pharmaceutical supply chain, it is important to have information flow from end- users to production site in order to recognize which and how many products should be planned (51-53), but 17% of the respondents presented that they have never been aware of market research and 31% claimed that they have feedback from customers needs by marketing department reports. 

## Conclusion

Due to recent rapid growth of imported medicine in Iran (15), it is so important to take a suitable strategy in both national drug policy and within of a company in order to retain our domestic industry thrive in the future. Furthermore, regarding to increasing novel drugs price as a result of huge investment on product development in the world (54), if they would be affordable to patients, it is necessary to allocate a large volume of resource to purchase them, while it is not accepted economically or even impossible especially for developing countries like Iran with low level of resources which allocated to health system.
